# Exosomal lncRNA Nuclear Paraspeckle Assembly Transcript 1 (NEAT1)contributes to the progression of allergic rhinitis via modulating microRNA-511/Nuclear Receptor Subfamily 4 Group A Member 2 (NR4A2) axis

**DOI:** 10.1080/21655979.2021.1982313

**Published:** 2021-10-21

**Authors:** Tao Wang, Weiyu Cai, Qinwei Wu, Dong Chen, Peihua Wang, Zhou Xu

**Affiliations:** Department of Otolaryngology Head & Neck Surgery, Shanghai Ninth People’s Hospital, School of Medicine, Shanghai Jiao Tong University, Shanghai, China

**Keywords:** Allergic rhinitis, NEAT1, miR-511, NR4A2, interleukin-13

## Abstract

Allergic rhinitis (AR) is a common chronic disease characterized by inflammation of the nasal mucosa. Long non-coding RNA (LncRNA) has been reported to be involved in the pathogenesis of various diseases. However, the biological roles of lncRNA Nuclear Paraspeckle Assembly Transcript 1 (NEAT1) in AR are still unclear. The mRNA levels of NEAT1, miR-511, and Nuclear Receptor Subfamily 4 Group A Member 2 (NR4A2) were detected by RT-qPCR. The protein levels of exosomal markers were examined by western blot. ELISA was used to assess the levels of GM-CSF, eotaxin-1, and MUC5AC. The cell viability and apoptosis were evaluated by CCK-8 and TUNEL assays. In this study, we found that the NEAT1 level was highly expressed in AR and IL-13-treated HNECs. NEAT1 interference significantly suppressed levels of GM-CSF, eotaxin-1, and MUC5AC and apoptosis rate, but promoted the viability of IL-13-treated human nasal epithelial cells (HNECs). Moreover, exosomes containing NEAT1 induced inflammatory cytokine production and apoptosis, while NEAT1 depletion abrogated these effects. In addition, NEAT1 directly interacted with miR-511, and the inhibition of miR-511 partially restored the inhibitory effects of NEAT1 silencing on inflammatory cytokine, mucus production, and apoptosis in IL-13-stimulated HNECs. Furthermore, miR-511 could bind to the 3ʹUTR of NR4A2, and the inhibition of miR-511 increased levels of inflammatory factors and apoptosis rate, which was counteracted by depleting NR4A2. In conclusion, our data revealed that exosomal NEAT1 contributed to the pathogenesis of AR through the miR-511/NR4A2 axis. These findings might offer novel strategies for the prevention and treatment of AR.

## Introduction

Allergic rhinitis (AR), a common chronic inflammatory disorder, is characterized by the symptoms of pruritus, sneezing, rhinorrhea, and nasal congestion [1]. Even though AR is not a life-threatening disease, it can reduce people’s quality of life and has a negative impact on the social economy [2]. Therefore, it is important to understand the molecular mechanisms underlying the pathogenesis of AR and develop novel effective therapeutic approaches for AR.

Long non-coding RNAs (lncRNAs) are a group of endogenous RNA molecules with > 200 nucleotides in length and lacking protein-coding ability [3,4]. Increasing evidence indicated that dysregulation of lncRNA participated in the development of inflammation-related diseases, including AR. For instance, lncRNA ANRIL was reported to be implicated in AR pathogenesis, and ANRIL level was associated with the risk, severity, and increased inflammation of AR [5]. Linc00632 suppressed IL-13-induced mucus production and inflammatory cytokine through regulating miR-498 in AR [6]. NEAT1 has also been confirmed to play a vital role in inflammatory diseases. For example, NEAT1 promoted inflammatory response in sepsis-induced liver injury through the Let-7a/TLR4 axis [7]. Knockdown of NEAT1 inhibited inflammatory response in LPS-induced acute injury via HMGB1-RAGE pathway [8]. Moreover, Wang et al reported that NEAT1 was highly expressed in AR and presented positive correlations with severity and inflammation of AR [9]. However, the exact function of NEAT1 in AR remains unknown.

Exosomes are membrane-derived vesicles, which contain various biomolecules, secreted from their parental cell cytoplasm, and may be absorbed into recipient cells. Increasing researches indicate that exosomes are involved in cellular communication and multiple physiological processes, including inflammation [10]. For example, Meng et al implied that exosome-mediated lncRNA PVT1 modulated LPS-triggered osteoarthritis development via regulating miR-93-5p [11]. Song et al demonstrated that adipocyte-derived exosomes-mediated SNHG9 inhibited inflammation and apoptosis of endothelial cells via downregulating TRADD [12]. Previous studies indicated that exosomal NEAT1 acted as a vital regulator in the pathogenesis of various diseases, such as autoimmune disorders [13], malignancies [14], and cardiovascular diseases [15]. Nevertheless, the involvement of exosome-mediated transfer of NEAT1 in AR is unclear.

In our study, we aimed to explore the function and molecular mechanism of NEAT1 in AR, and we hypothesized that NEAT1 contributed to the pathogenesis of AR. Our results demonstrated that exosomal NEAT1 promoted AR development through miR-511/NR4A2 axis. These discoveries might provide a promising treatment strategy for patients with AR.

## Materials and methods

### Clinical samples

Nasal mucosal samples were collected from inferior turbinate mucosa from 30 patients with perennial AR and 30 age- and sex-matched healthy controls using a nasal endoscopy [16]. No patients received topical or systemic corticosteroid therapy prior to the first 4 weeks of enrollment. Written informed consent was provided from all participators before the start of the study. This study was approved by the Ethics Committee of the Shanghai Ninth People’s Hospital.

### Assessment of AR severity

The severity of AR patients was evaluated by sneezing, rhinorrhea, nasal itching, and congestion, and each item was scored as 0 (no symptoms), 1 (mild symptoms), 2  (moderate symptoms), and 3 (severe symptoms) [5]. Then, the total nasal symptom score (TNSS) was calculated by summing the scores of the above items.

### Cell culture and treatment

Primary human nasal epithelial cells (HNECs) were purchased from iCell Bioscience (Shanghai, China), and cultured in RPMI-1640 medium (Gibco, USA) supplemented with 10% FBS (Gibco, USA), 1% penicillin (Invitrogen, USA) and 1% streptomycin (Invitrogen, USA) maintained at 37°C and 5% CO_2_. 50 ng/ml IL-13 (Sigma-Aldrich, USA) was used to treat HNECs for 24 h to establish an in vitro model of AR as described previously [17].

### Cell transfection

Small interfering RNAs (siRNAs) targeting NEAT1 (si-NEAT1) and NR4A2 (si-NR4A2) with their negative control (si-NC), and miR-511 mimics and miR-511 inhibitor with their negative control (NC mimic and NC inhibitor) were obtained from GenePharma (Shanghai, China). HNECs (1 x 10^5^) were transfected with si-NEAT1, si-NR4A2, si-NC, miR-511, NC mimics, miR-511 inhibitor or NC inhibitor using Lipofectamine 2000 (Invitrogen, USA). HNECs were collected 48 h after transfection according to previous study [18].

### ELISA

GM-CSF and eotaxin-1 in the cell culture supernatants were examined by ELISA kits from R&D Systems (Minneapolis, USA), and MUC5AC was measured by Human Mucin-5 subtype AC ELISA kit from BluegGene Biotech (Shanghai, China) according to manufacturer’s protocols.

### TUNEL assay

An in-situ Cell Death Detection Kit was utilized to evaluate the apoptosis rate of HNECs [19]. Briefly, HNECs cells were washed with PBS (Thermo Fisher Scientific, USA) and fixed in paraformaldehyde (Sigma-Aldrich, USA). Next, the cells were treated with 0.1% Triton X-100 (Thermo Fisher Scientific, USA) for 2 min and cultured with TUNEL reaction mixture at 37°C for 1 h. Subsequently, the TUNEL-stained cells were counterstained with DAPI. Apoptotic cells were detected by a fluorescence microscope.

### Cell viability assay

The transfected cells were seeded into 96-well plates at a density of 5 × 10^3^ cells/well and incubated for 0, 24, 48, 72 h. Subsequently, 10 μL CCK-8 solution was added to each well and incubated for 4 h at 37°C. Cell viability was assessed by a microplate reader at 450 nm [18].

### Luciferase reporter assays

Wild-type or mutant NEAT1 (or NR4A2) sequence were subcloned into pmirGLO vector (Promega) to establish NEAT1-WT (NR4A2-WT) or NEAT1-Mut (NR4A2-Mut) reporter vectors. Mutants within the miR-511 binding site were created using the QuikChange II Site-Directed Mutagenesis kit (Agilent Technologies, Inc., USA). Then, the aforementioned vectors were co-transfected with miR-511 mimic or NC mimic into HNECs. The luciferase activity was detected by a dual-luciferase reporter system (Promega, USA) [20].

### RT-qPCR

Total RNA was extracted from nasal mucosal samples and HNECs using TRIzol Reagent (Invitrogen, USA). cDNA was synthesized using PrimeScript RT Reagent kit (Takara, China). RT-qPCR was conducted with the ABI 7900 Detection System (Applied Biosystems) using the SYBR-Green PCR Master Mix kit (Takara Bio, Inc). GAPDH and U6 were used as internal controls.

### Western blot

Total proteins were extracted using RIPA buffer (Invitrogen, USA). Proteins (20 μg) were separated using 10% SDS-PAGE and transferred to a PVDF membrane (EMD Millipore, USA). The membranes were blocked with 5% skimmed milk and incubated with primary antibodies against CD9, CD63, or GAPDH at 4°C overnight. Subsequently, the membranes were incubated with secondary antibodies for 1 h. Then, the bands were evaluated with the enhanced chemiluminescence (ECL) Kit (Pierce, Thermo Fisher Scientific, IL, USA).

### Exosome isolation and identification

Exosomes were extracted from the cell culture medium using an ExoQuick precipitation kit (System Biosciences, USA) [21]. The culture medium was collected and centrifuged at 3,000 x g for 15 min to remove cells and cell debris. Next, 250 μl supernatant was mixed with 63 μl ExoQuick precipitation kit and incubated at 4°C for 30 min, followed by centrifugation at 1,500 × g for 30 min at 4°C. The supernatant was removed via careful aspiration, followed by another 5 min of centrifugation to remove the residual liquid. The exosome pellet was subsequently resuspended in 200 μl PBS. Exosome markers CD9 and CD63 were verified by western blot analysis, and exosomal morphology was observed by a transmission electron microscope (TEM; JEM1010, JEOL, Japan).

### Statistical analysis

The experiments were repeated in triplicate. Statistical analysis was performed using SPSS 23.0 software (SPSS, Inc., USA) and the results are presented as the mean ± SD. Statistical differences were analyzed using Student’s t-test or one-way analysis of variance (ANOVA). P < 0.05 was considered statistically significant.

## Results

### NEAT1 is highly expressed in AR

**Figure 1.**
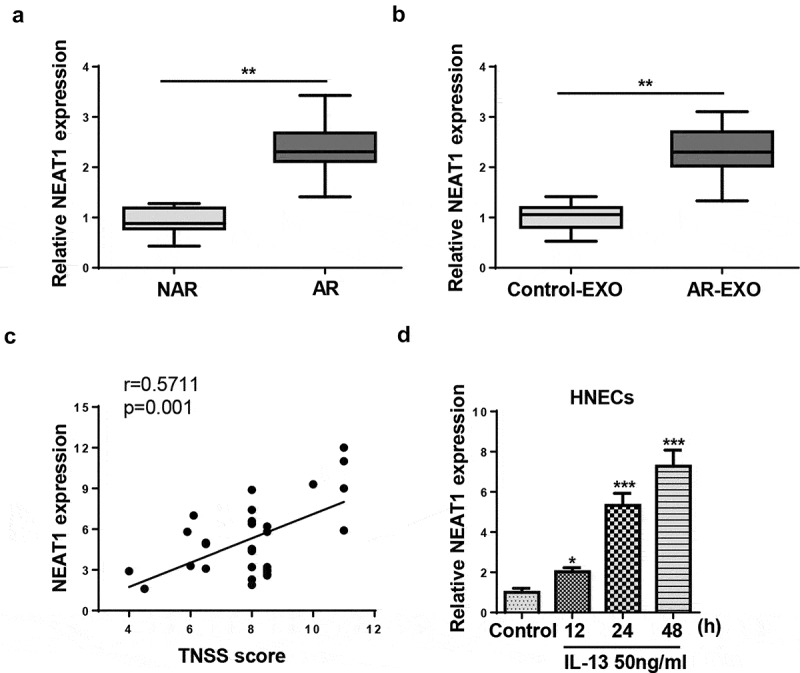
NEAT1 expression is elevated in nasal mucosal tissues from AR patients and positively correlated to IL-13 stimulation. (a) RT-qPCR showed NEAT1 expression levels in mucosal tissues from 30 patients with perennial AR and 30 patients with nonallergic rhinitis (NAR) were measured. (b) RT-qPCR showed NEAT1 expression in the AR-EXO and control-EXO. (c) Correlation of lncRNA NEAT1 expression with TNSS score. (d) RT-qPCR showed NEAT1 expression in human HNECs treated with 50 ng/mL IL-13 for 12, 24, or 48 h. *P < 0.05, **P < 0.01, ***P < 0.001

To explore the expression pattern of NEAT1 in AR, we examined NEAT1 levels in the nasal mucosa of patients with AR. RT-qPCR results indicated that NEAT1 expression was enhanced in AR patients compared with non-AR patients (Figure 1(a)). In addition, the exosomes were extracted from nasal mucus samples of AR patients (AR-EXO) and healthy controls (control-EXO), and the results indicated that NEAT1 expression was highly expressed in AR-EXO compared with that in control-EXO (Figure 1(b)). Moreover, NEAT1 expression was positively correlated with TNSS score, indicating that NEAT1 was associated with the severity of AR (Figure 1(c)). IL-13 was reported to promote mucus production and the secretion of inflammatory cytokines in nasal epithelial cells [22]. Therefore, IL-13 treated HNECs were used to establish AR cell model. RT-qPCR analysis showed that NEAT1 levels were upregulated with IL-13 stimulation (Figure 1(d)). These results indicated that NEAT1 was upregulated in patients with AR and IL-13-treated HNECs.

### NEAT1 knockdown regulates IL-13-triggered inflammatory cytokine, mucus production, and apoptosis in HNECs

**Figure 2.**
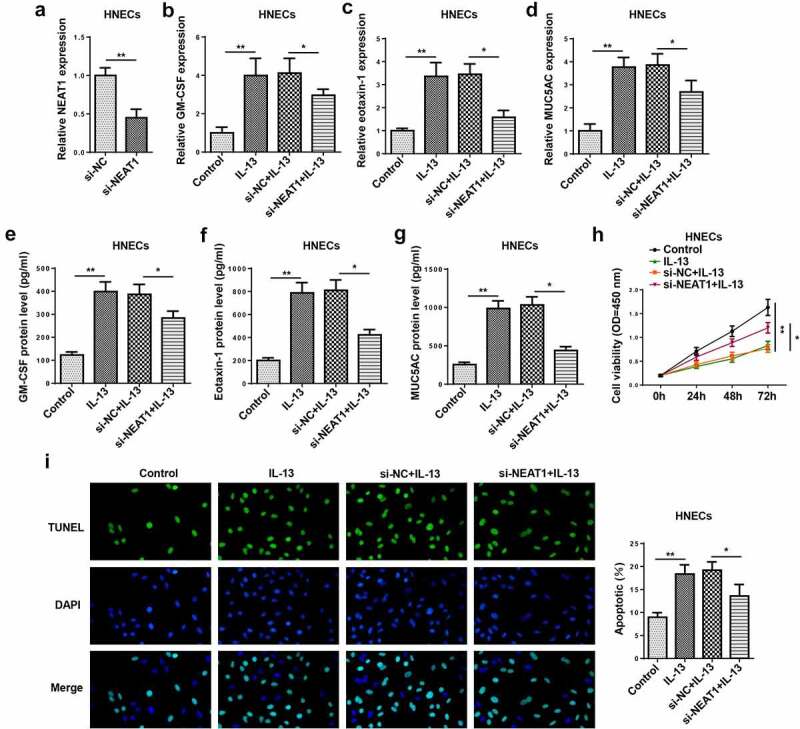
NEAT1 knockdown regulates IL-13-triggered inflammatory cytokine, mucus production, and apoptosis in HNECs. (a). RT-qPCR showed the level of NEAT1 in human HNECstransfected with si-NEAT1 or si-NC. (b–g) RT-qPCR and ELISA assay showed the expression levels of GM-CSF, eotaxin-1, and MUC5AC in IL-13-stimulated HNECs transfected with si-NEAT1 or si-NC. (h and i) CCK-8 and TUNEL assays indicated the cell viability and apoptosis in IL-13-treated HNECs transfected with si-NEAT1 or si-NC. *P < 0.05, **P < 0.01

To determine the function of NEAT1 in HNECs, HNECs were transfected with si-NC or si-NEAT1. The knockdown efficiency was confirmed by RT-qPCR (Figure 2(a)). Moreover, RT-qPCR and ELISA indicated that the mRNA and protein levels of GM-CSF, eotaxin-1, and MUC5AC were enhanced in IL-13-induced HNECs, while NEAT1 knockdown reversed these effects (Figure 2(b–g)). In addition, NEAT1 silencing significantly promoted the viability (Figure 2(h)), and suppressed the apoptosis of IL-13-treated HNECs (Figure 2(h)). These data determined that NEAT1 knockdown restrained IL-13-stimulated inflammatory cytokine and mucus production levels in HNECs.

### Extracellular NEAT1 is transferred via incorporation in exosomes in HNECs

**Figure 3.**
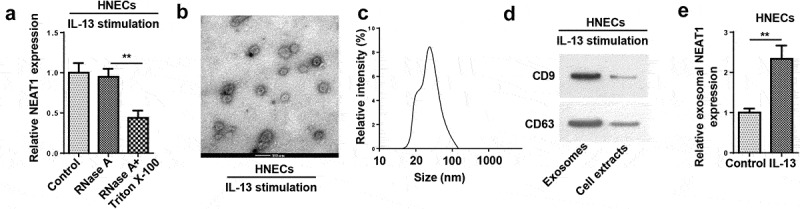
Extracellular NEAT1 was transferred via incorporation in exosomes in HNECs. (a) The expression of NEAT1 was detected by RT-qPCR after cells were treated with RNase A or RNase A + 0.1% Triton X100 for 30 min. (b) The exosomes images secreted by IL-13-treated HNECs were showed by TEM scanning. (c) Size distribution of exosomes ranged from 30 to 120 nm. (d) The levels of exosomal marker proteins CD9 and CD63 were measured by Western blot in HNECs. (e) RT-qPCR analysis showed the expression of NEAT1 in exosomes extracted from IL-13-treated HNECs cells. **P < 0.01

Subsequently, we explored the effect of exosomal NEAT1 on the progression of AR. NEAT1 level was evaluated after treatment with RNase A or RNase A+Triton X-100. RT-qPCR analysis displayed that NEAT1 expression was non significantly changed after RNase A treatment, but RNase A and Triton X-100 treatment simultaneously showed an obvious reduction in NEAT1 level (Figure 3(a)). Subsequently, the representative micrograph obtained by TEM exhibited a typical lipid bilayer membrane (Figure 3(b)), and the exosome particle diameters ranged from 30 to 120 nm (Figure 3(c)). Then western blot assay elaborated that the levels of exosomal markers CD9 and CD63 were enriched in exosomes (Figure 3(d)). Besides, RT-qPCR analysis implied that NEAT1 level was elevated in exosomes extracted from IL-13-treated HNECs cells (Figure 3(e)). Altogether, these data manifested that extracellular NEAT1 was secreted by packaging into exosomes of HNECs.

### NEAT1 silence attenuates the exosome-induced inflammatory response and apoptosis of HNECs

**Figure 4.**
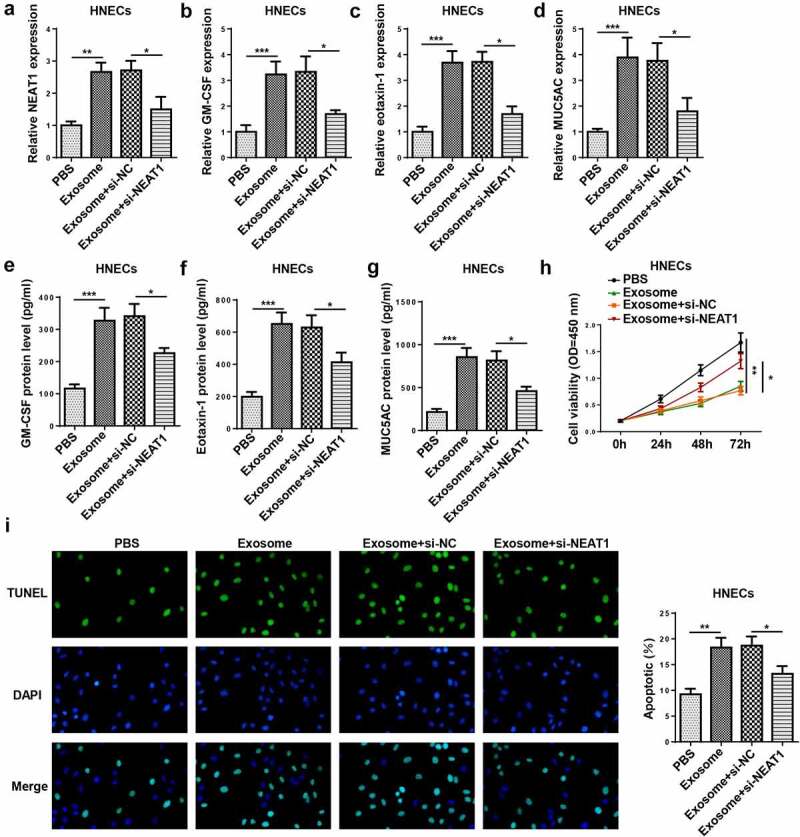
NEAT1 silence attenuates the exosome-induced inflammatory response and apoptosis of HNECs. (a) RT-qPCR showed NEAT1 level in HNECs treated with PBS, exosome, exosome+si-NC, and exosome+si-NEAT1. (b–g) RT-qPCR and ELISA assay showed the mRNA expression levels of GM-CSF, eotaxin-1, and MUC5AC in HNECs treated with exosome, exosome+si-NC, and exosome+si-NEAT1. (h and i) CCK-8 and TUNEL assays indicated cell viability and apoptosis in different groups. *P < 0.05, **P < 0.01, ***P < 0.001

To study the involvement of exosomal NEAT1 in the pathogenesis of AR, HNECs were treated with exosomes which were extracted from IL-13-treated HNECs culture medium or transfected with si-NEAT1. NEAT1 level was elevated by exosome treatment and reduced by NEAT1 knockdown in HNECs cells (Figure 4(a)). Moreover, the mRNA and protein levels of GM-CSF, eotaxin-1, and MUC5AC were elevated in HNECs treated with exosomes, which was decreased by NEAT1 depletion (Figure 4(b–g)). In addition, CCK-8 and TUNEL assays revealed that exosome treatment inhibited cell viability and promoted apoptosis, while these effects could be reversed by NEAT1 silencing (Figure 4(h,i)). Thus, we verified that exosome conferred inflammatory response and apoptosis via upregulating NEAT1 in HNECs.

### NEAT1 acts as a molecule sponge for miR-511

**Figure 5.**
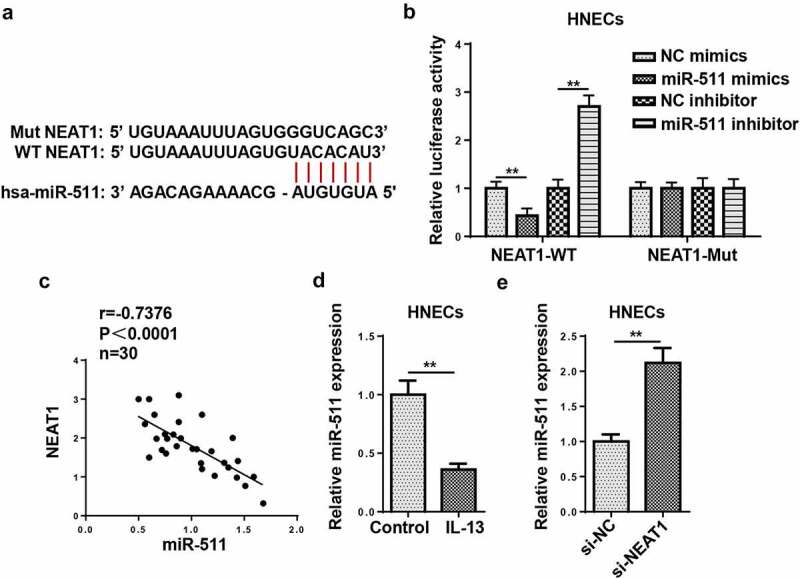
NEAT1 acts as a molecule sponge for miR-511. (a) The putative binding sites between NEAT1 and miR-511 were predicted by starBase website. (b) The luciferase activity of NEAT1-WT and NEAT1-Mut were measured in HNECs transfected with NC mimics, miR-511 mimics, NC inhibitor, or miR-511 inhibitor. (c) Correlation analysis between miR-511 and NEAT1 in nasal mucosal tissues from AR patients. (d) The expression of miR-511 was detected by RT-qPCR. (e) RT-qPCR showed miR-511 expression in HNECs transfected with si-NEAT1 or si-NC. **P < 0.01

Bioinformatics prediction revealed that NEAT1 could potentially bind to miR-511 (Figure 5(a)). Luciferase reporter assay indicated that miR-511 overexpression suppressed the luciferase activity of NEAT1-WT, but there was no distinct change in NEAT1-Mut group (Figure 5(b)). Besides, NEAT1 levels were inversely correlated with miR-511 levels (Figure 5(c)). Subsequently, RT-qPCR indicated that miR-511 expression was declined in IL-13-treated HNECs (Figure 5(d)). The silencing of NEAT1 elevated miR-511 expression in HNECs (Figure 5(e)). These data implied that NEAT1 could negatively regulate miR-511 expression by direct interaction.

### miR-511 is a downstream regulator of NEAT1 in AR

**Figure 6.**
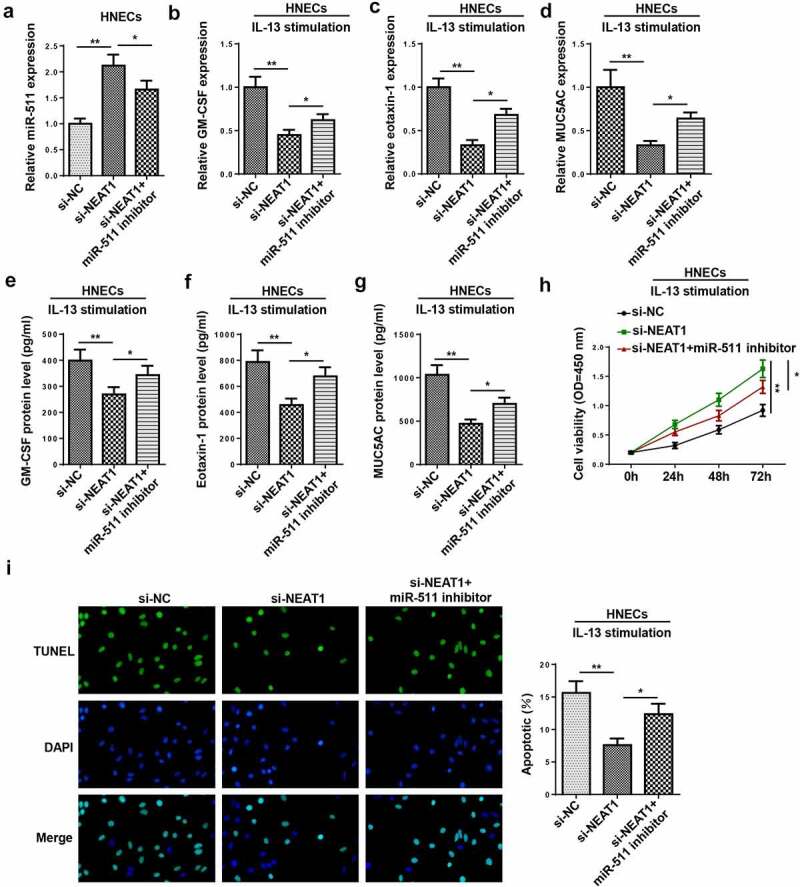
miR-511 is a downstream regulator of NEAT1 in AR. (a) RT-qPCR showed miR-511 expression in HNECs transfected with si-NC, si-NEAT1, and si-NEAT1+miR-511 inhibitor. (b–g) RT-qPCR and ELISA assay showed the expression levels of GM-CSF, eotaxin-1, and MUC5AC in IL-13-treated HNECs transfected with si-NC, si-NEAT1, and si-NEAT1+ miR-511 inhibitor. (h and i) CCK-8 and TUNEL assays indicated the cell viability and apoptosis in IL-13-treated HNECs transfected with si-NC, si-NEAT1, and si-NEAT1+ miR-511 inhibitor. *P < 0.05, **P < 0.01

To further investigate whether miR-511 participated in NEAT1-mediated AR progression, rescue assays were performed. Results implied that the interference of NEAT1 increased the miR-511 level, which was counteracted by miR-511 inhibition (Figure 6(a)). Moreover, miR-511 suppression rescued the repressive effects of NEAT1 interference on inflammatory cytokine and mucus production in IL-13-treated HNECs (Figure 6(b–g)). In addition, the inhibition of miR-511 abolished the effects of NEAT1 knockdown on the viability and apoptosis of IL-13-induced HNECs (Figure 6(h,i)). In sum, these discoveries revealed that NEAT1 regulated the levels of inflammatory factors by targeting miR-511.

### NR4A2 is a target of miR-511

**Figure 7.**
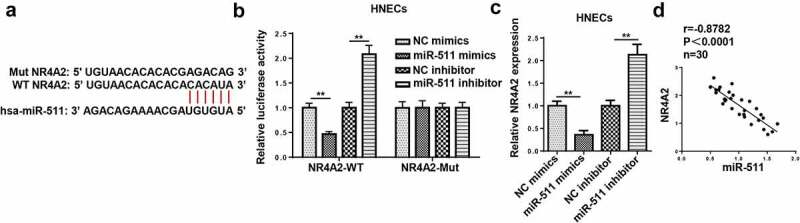
NR4A2 is a target of miR-511. (a) The putative binding sites between NR4A2 and miR-511 were predicted by TargetScan. (b) The luciferase activity of NR4A2-WT and NR4A2-Mut were measured in HNECs cells transfected with NC mimics or miR-511 mimics. (c) RT-qPCR showed NR4A2 expression in HNECs transfected with NC mimics, miR-511 mimics, NC inhibitor, or miR-511 inhibitor. (d) Correlation analysis between miR-511 and NR4A2 in nasal mucosal tissues from AR patients. **P < 0.01

According to the prediction of TargetScan, we found that 3ʹ-UTR of NR4A2 had a binding site for miR-511 (Figure 7(a)). As shown in Figure 7(b), overexpression of miR-511 decreased the luciferase activity of NR4A2-WT, while there was no alteration in NR4A2-Mut. Furthermore, the upregulation of miR-511 decreased NR4A2 expression, and the downregulation of miR-511 increased NR4A2 expression (Figure 7(c)). Furthermore, NR4A2 expression was negatively correlated with miR-511 expression (Figure 7(d)). The above data elucidated that miR-511 could bind to the 3ʹ UTR of NR4A2.

### NEAT1 regulates IL-13-induced dysfunction of HNECs via miR-511/NR4A2 axis

**Figure 8.**
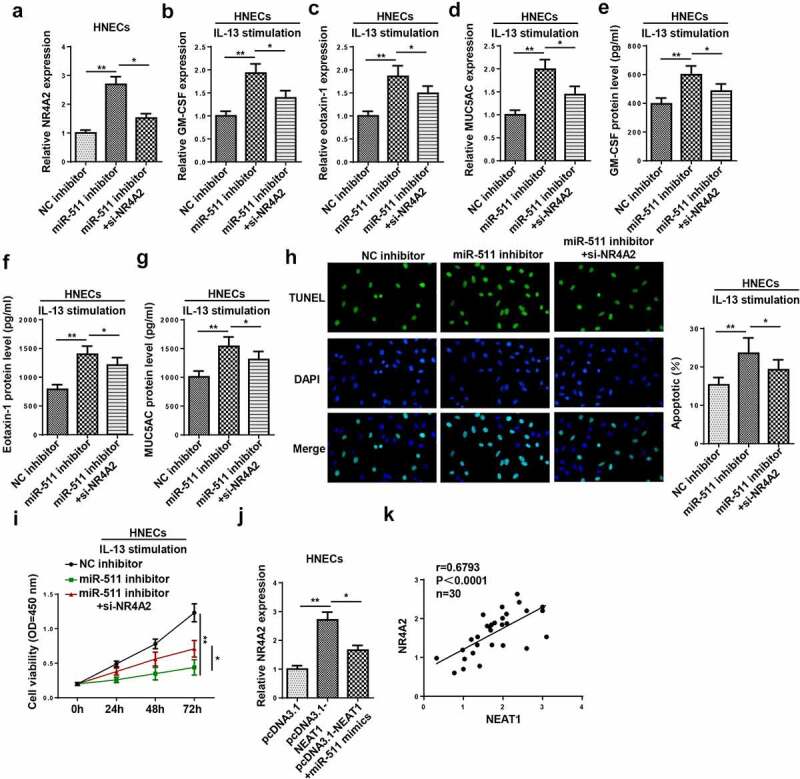
NEAT1 regulates IL-13-induced dysfunction of HNECs via miR-511/NR4A2 axis. (a) NR4A2 expression was detected by RT-qPCR in HNECs transfected with NC inhibitor, miR-511 inhibitor, miR-511 inhibitor+si-NR4A2. (b–g) The levels of GM-CSF, eotaxin-1, and MUC5AC were determined by RT-qPCR and ELISA in IL-13-treated HNECs transfected with NC inhibitor, miR-511 inhibitor, miR-511 inhibitor + si-NR4A2. (h and i) TUNEL and CCK-8 assays indicated the cell apoptosis and viability in IL-13-treated HNECs transfected with NC inhibitor, miR-511 inhibitor, miR-511 inhibitor + si-NR4A2. (j) NR4A2 expression was measured by RT-qPCR in HNECs transfected with pcDNA3.1, pcDNA3.1-NEAT1, pcDNA3.1-NEAT1+ miR-511 mimics. (k) Correlation analysis between NEAT1 and NR4A2 in nasal mucosal tissues from AR patients. *P < 0.05, **P < 0.01

To explore whether NR4A2 was an important effector of the NEAT1/miR-511 axis in AR, NC inhibitor, miR-511 inhibitor, and miR-511 inhibitor+NR4A2 were transfected into HNECs. As exhibited in Figure 8(a), the inhibition of miR-511 increased NR4A2 expression, which was decreased by NR4A2 silencing. Moreover, the inhibition of miR-511 promoted inflammatory cytokine and mucus production in IL-13-induced HNECs, while these effects were abrogated by NR4A2 downregulation (Figure 8(b–g)). The downregulation of NR4A2 reversed miR-511 silencing-mediated the effects on apoptosis and viability of IL-13-treated HNECs (Figure 8(h,i)). In addition, the upregulation of miR-511 abolished the promoting effect of NEAT1 overexpression on NR4A2 expression (Figure 8(j)), and the expression of NEAT1 was positively correlated with NR4A2 expression (Figure 8(k)). Therefore, our data suggested that NEAT1 might regulate AR progression via the miR-511/NR4A2 axis.

## Discussion

Growing evidence indicated that NEAT1 participated in inflammatory activities of several diseases [23,24]. For instance, NEAT1 promoted the activation of inflammasomes in macrophages [25]. NEAT1 facilitated inflammatory injury and apoptosis in pneumonia through TLR4/NF-κB signaling [26]. A previous study indicated that NEAT1 was upregulated in AR and positively correlated with inflammatory cytokines and nasal symptom scores (rhinorrhea, itching, congestion scores) [9]. Consistently, in our study, we uncovered that NEAT1 was highly expressed in AR. Loss-of-function assays revealed that NEAT1 silencing restrained IL-13-triggered inflammatory response and apoptosis in HNECs. Multiple studies have determined that exosome can act as a mediators of the inflammatory response in various diseases, such as inflammatory bowel diseases [27], neurodegenerative diseases [28], and atherosclerosis [29]. In addition, NEAT1 could be secreted by the exosome to participate in the inflammatory response of human diseases. For example, NEAT1 shuttled by PBMC-derived exosome promoted fibroblast-like synoviocytes proliferation and inflammation via the MDM2/SIRT6 pathway [30]. Exosomal NEAT1 protected against doxorubicin-induced cardiac senescence by regulating miR-221-3p [31]. Herein, we uncovered that NEAT1 could be packaged in exosomes, and knockdown of NEAT1 could relieve exosome-induced inflammatory response and apoptosis of HNECs.

It is widely reported that lncRNAs can act as molecular sponges for miRNAs to regulate the pathological process of diseases. For instance, lncRNA IGHCγ1 served as a ceRNA to modulate macrophage inflammation in osteoarthritis by targeting miR-6891-3p and modulating TLR4 expression [32]. MEG3 facilitated inflammatory response and fibrosis by regulating the miR-181a/Egr-1/TLR4 axis in diabetic nephropathy [33]. Moreover, miRNAs could act as a vital regulator in the pathogenesis of AR. miR-143 repressed IL-13-triggered inflammatory cytokine via regulating IL13Rα1 in AR [17]. MiR-487b improved AR through suppressing the IL-33/ST2 signaling pathway [34]. miR-511 was uncovered to exhibit an inhibitory effect on allergic inflammation [35]. Here, we found that miR-511 was a downstream target of NEAT1. In addition, the silencing of NEAT1 suppressed inflammatory response and apoptosis in IL-13-treated HNECs, which was reversed following miR-511 inhibition.

NR4A2, a member of the NR4A orphan nucleus receptor family [36], has been identified as a transcriptional activator of IL-8 in human inflammatory arthritis [37], and orchestrates Th17 cell-mediated autoimmune inflammation through IL-21 signaling [38]. In this study, we found that miR-511 could directly bind to NR4A2. Moreover, the downregulation of miR-511 promoted the levels of inflammatory factors and apoptosis in IL-13-induced HNECs, while the knockdown of NR4A2 reversed these effects. Furthermore, miR-511 mimics restored NEAT1 overexpression-mediated stimulative effects on NR4A2 expression. These results indicated that exosomal NEAT1 acted as a ceRNA in regulating NR4A2 through sponging miR-511 in AR.

## Conclusion

Our study demonstrated that exosomal NEAT1 silencing suppressed IL-13-induced inflammatory cytokine, mucus production, and cell apoptosis in AR via miR-511/NR4A2 axis. These findings suggested that NEAT1 might be an effective target for patients with AR. In the future study, in vivo experiments should be performed to further confirm the role and function of NEAT1 in AR.
